# Effects of radiotherapy on the survival of patients with malignant spermatic cord tumors: A retrospective cohort study

**DOI:** 10.1002/cam4.5402

**Published:** 2022-11-10

**Authors:** Yifu Liu, Zhicheng Zhang, Jinxiang Wang, Siyuan Wang

**Affiliations:** ^1^ Department of Urology The First Affiliated Hospital of Nanchang University Nanchang Jiangxi China; ^2^ Department of Cell Biology, School of Basic Medical Sciences Southern Medical University Guangzhou China; ^3^ Department of Urology Sichuan Cancer Hospital Chengdu China

**Keywords:** radiotherapy, spermatic cord tumor, survival

## Abstract

**Background:**

Malignant spermatic cord tumors (SCT) are rare and currently, there is no consensus regarding the role of radiotherapy in their treatment. This study evaluated the effect of radiotherapy on the overall survival (OS) of patients with malignant SCT based on the large‐sample advantage of the Surveillance, Epidemiology, and End Results (SEER) database.

**Methods:**

Patients with malignant SCT recorded in the SEER database from 1975 to 2016 were included. All patients were divided into non‐radiation and radiation groups, and propensity score matching (PSM) (1:1) was performed for baseline covariates between the two groups. The overall survival rate between the two groups of patients was analyzed using the Kaplan–Meier curve. The effects of radiotherapy on patient prognosis were analyzed using univariate and multivariate COX regression analyses.

**Results:**

In total, 389 patients with malignant SCT were included. There were 285 (73.26%) and 104 (26.74%) patients who either did or did not receive radiotherapy, respectively. Kaplan–Meier curves before and after PSM showed no significant differences in OS between the two groups. Similarly, multivariate COX regression models before and after PSM showed that radiotherapy was not an independent risk factor for OS in patients with malignant SCT.

**Conclusions:**

Radiotherapy has no obvious advantage in improving the survival time of patients with malignant SCT.

## 1 INTRODUCTION

Spermatic cord tumors (SCT) are the most common paratesticular tumors,^1^ with a malignancy rate of 31%.^2^ The annual incidence of malignant SCT is 0.3 cases per million people,^3^ most of which are primary tumors, and distant metastasis is rare.^4^ The most common histological types of malignant spermatic tumors include liposarcoma, leiomyosarcoma, rhabdomyosarcoma, malignant fibrous histiocytoma, and fibrosarcoma.^5^

Coleman et al.^6^ in 2003 reported that the median age of adult patients with spermatic cord sarcoma is 58 years. The median follow‐up period was 51 months and the overall 5‐ and 10‐year disease‐specific survival rates were 75% and 55%, respectively. Similarly, Radaelli et al.^7^ in 2014 reported a median follow‐up of 33 months for patients with spermatic cord sarcoma. The 5‐year disease‐specific survival, local recurrence, and distant metastasis rates were 92% (95% confidence interval CI 83–97), 26% (95% CI 15–42), and 24% (95% CI 15–38), respectively.

The current consensus is that surgical intervention is the gold standard treatment for SCT. Radical resection, wide tumor resection with ipsilateral scrotal resection, and high spermatic cord ligation are commonly used.^8^ The role of radiotherapy and chemotherapy in the treatment of SCT remains uncertain.^9^ In this study, we used the Surveillance, Epidemiology, and End Results (SEER) database's dominance in rare tumor case numbers to determine whether radiation therapy improved the overall survival (OS) in patients with SCT.

## 2 METHODS

### 2.1 Patients

The data for our retrospective study were all obtained from the SEER database (https://seer.cancer.gov/). We selected the data set from 1975 to 2016 on SEER*Stat software (version 8.3.9.2) and used histological type ICD‐O Code C63.1 to collect clinicopathological, treatment, and follow‐up data of patients with malignant SCT.

**FIGURE 1:**
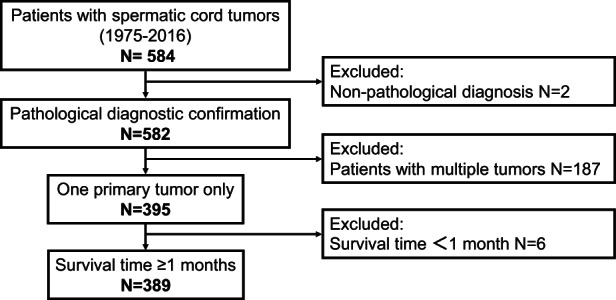
Schematic representation of the inclusion and exclusion criteria for the study cohort

A total of 584 patients with SCT were identified. The following exclusion criteria were used for data screening: no pathological diagnosis (*n* = 2), multiple tumors (*n* = 187), and survival time of less than 1 month (*n* = 6). Finally, 389 eligible patients with SCT were included in the study (Figure 1).

### 2.2 Statistical analysis

All statistical calculations were performed using EmPowerStats (http://www.empower stats.com; X&Y Solutions, Boston, MA, USA) and R statistical package (http://www.r‐project.org). A two‐sided *p* < 0.05 was considered statistically significant. Baseline data, including demographic and clinical characteristics were expressed as frequencies and component ratios. To explore the effect of radiation therapy on OS, while excluding the effects of other variables, we performed propensity score matching (PSM) analysis in patients with or without radiation therapy. The 1:1 PSM matched patients according to the following factors: year of diagnosis, age at diagnosis, race, marital status, laterality, tumor size, histology, grade, stage, regional nodes examined, regional nodes positive, chemotherapy and surgery, using the nearest‐neighbor matching method in the matching strategy, with a caliper width of 0.02. OS was assessed using the Kaplan–Meier method. Prognostic factors associated with OS were performed using Cox proportional hazards models, with results expressed as hazard ratios (HR) and 95% confidence intervals (95% CI). In univariate analysis, variables with *p*‐values less than 0.05 and the receipt of radiotherapy were included as candidate variables in the multivariate analysis.

## 3 RESULTS

### 3.1 Characteristics of patients with SCT

Of the 389 SCT patients who met the inclusion criteria, 285 (73.3%) did not receive radiation therapy and 104 (26.7%) received radiation therapy. There were differences in the years of diagnosis, pathological grades, and surgical methods between the two groups (*p* < 0.05). After PSM, 98 pairs of patients were matched successfully. Baseline features were well balanced in the matched cohorts. Table 1 shows the baseline characteristics of the two groups of patients before and after PSM.

**TABLE 1:** Baseline characteristics of SCT patients

Characteristic	No. of patients before PSM (%)			No. of patients after PSM (%)		
	No Radiation group	Radiation group		No Radiation group	Radiation group	
	(*n* = 285)	(*n* = 104)		(*n* = 98)	(*n* = 98)	*p*‐value
Year of diagnosis			0.005*			1
1975–1995	97 (34.04%)	18 (17.31%)		18 (18.37%)	18 (18.37%)	
1996–2006	78 (27.37%)	38 (36.54%)		34 (34.69%)	34 (34.69%)	
2007–2016	110 (38.60%)	48 (46.15%)		46 (46.94%)	46 (46.94%)	
Age at diagnosis			0.191			0.097
≤50	70 (24.56%)	19 (18.27%)		29 (29.59%)	19 (19.39%)	
>50	215 (75.44%)	85 (81.73%)		69 (70.41%)	79 (80.61%)	
Race			0.672			0.14
White	236 (82.81%)	88 (84.62%)		76 (77.55%)	84 (85.71%)	
Other	49 (17.19%)	16 (15.38%)		22 (22.45%)	14 (14.29%)	
Marital status			0.223			0.219
Married	202 (70.88%)	67 (64.42%)		71 (72.45%)	63 (64.29%)	
Other	83 (29.12%)	37 (35.58%)		27 (27.55%)	35 (35.71%)	
Laterality			0.472			0.782
Left	145 (50.88%)	47 (45.19%)		51 (52.04%)	46 (46.94%)	
Right	134 (47.02%)	56 (53.85%)		46 (46.94%)	51 (52.04%)	
Other	6 (2.11%)	1 (0.96%)		1 (1.02%)	1 (1.02%)	
Tumor size, mm			0.232			0.64
≤50	45 (15.79%)	18 (17.31%)		22 (22.45%)	17 (17.35%)	
>50	61 (21.40%)	30 (28.85%)		29 (29.59%)	29 (29.59%)	
Unknown	179 (62.81%)	56 (53.85%)		47 (47.96%)	52 (53.06%)	
Histology			0.502			0.836
Liposarcoma	153 (53.68%)	50 (48.08%)		50 (51.02%)	47 (47.96%)	
Leiomyosarcoma	50 (17.54%)	24 (23.08%)		22 (22.45%)	21 (21.43%)	
Histiocytoma	21 (7.37%)	11 (10.58%)		8 (8.16%)	11 (11.22%)	
Rhabdomyosarcoma	21 (7.37%)	8 (7.69%)		5 (5.10%)	8 (8.16%)	
Other	40 (14.04%)	11 (10.58%)		13 (13.27%)	11 (11.22%)	
Grade			**0.013** *			1
I	100 (35.09%)	24 (23.08%)		24 (24.49%)	24 (24.49%)	
II	34 (11.93%)	13 (12.50%)		13 (13.27%)	13 (13.27%)	
III	25 (8.77%)	19 (18.27%)		17 (17.35%)	17 (17.35%)	
IV	33 (11.58%)	19 (18.27%)		17 (17.35%)	17 (17.35%)	
Other	93 (32.63%)	29 (27.88%)		27 (27.55%)	27 (27.55%)	
Stage			0.448			0.973
Localized	189 (66.32%)	63 (60.58%)		64 (65.31%)	61 (62.24%)	
Regional	60 (21.05%)	30 (28.85%)		25 (25.51%)	27 (27.55%)	
Distant	13 (4.56%)	4 (3.85%)		3 (3.06%)	3 (3.06%)	
Other	23 (8.07%)	7 (6.73%)		6 (6.12%)	7 (7.14%)	
Regional nodes examined			0.634			0.281
No	194 (68.07%)	76 (73.08%)		73 (74.49%)	72 (73.47%)	
Yes	27 (9.47%)	8 (7.69%)		11 (11.22%)	6 (6.12%)	
Unknown	64 (22.46%)	20 (19.23%)		14 (14.29%)	20 (20.41%)	
Regional nodes positive			0.727			0.592
Negative	25 (8.77%)	8 (7.69%)		10 (10.20%)	7 (7.14%)	
Positive	3 (1.05%)	2 (1.92%)		2 (2.04%)	1 (1.02%)	
Unknown	257 (90.18%)	94 (90.38%)		86 (87.76%)	90 (91.84%)	
Chemotherapy			0.131			0.427
No/Unknown	250 (87.72%)	85 (81.73%)		85 (86.73%)	81 (82.65%)	
Yes	35 (12.28%)	19 (18.27%)		13 (13.27%)	17 (17.35%)	
Surgery			**0.002** *			1
Local tumor excision	48 (16.84%)	20 (19.23%)		18 (18.37%)	18 (18.37%)	
Radical surgery	125 (43.86%)	63 (60.58%)		61 (62.24%)	61 (62.24%)	
Other	112 (39.30%)	21 (20.19%)		19 (19.39%)	19 (19.39%)	

Abbreviations: No, Number; PSM, Propensity score matching; SCT, spermatic cord tumors.

* *p* < 0.05 was considered significant and marked in bold.

### 3.2 Survival analysis before and after PSM

**FIGURE 2:**
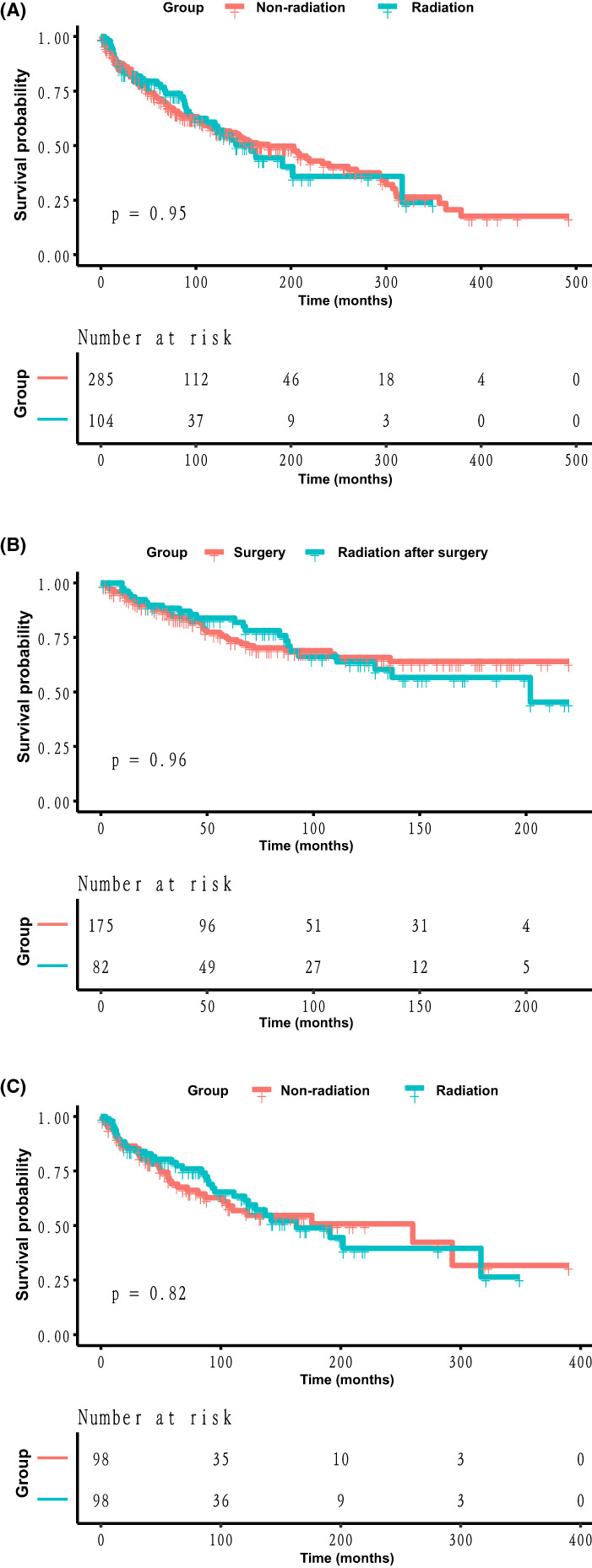
Kaplan–Meier curve analysis of the effect of radiotherapy on overall survival in patients with spermatic cord tumors (A) before and (C) after PSM. (B) Kaplan–Meier curves of patients in the two groups after surgery and radiation after surgery

We used Kaplan–Meier survival curves to compare OS between groups not receiving radiation therapy and those receiving radiation therapy. The results showed that there was no significant difference before PSM (Figure 2A) and after PSM (Figure 2C) (log‐rank *p* values were 0.95, 0.82, respectively). To determine whether radiotherapy would be of benefit to postoperative patients, we compared the survival curves between the two groups of patients who underwent surgery and postoperative radiotherapy. The results showed no significant difference between the two groups (log‐rank *p* = 0.96) (Figure 2B). Before PSM was applied, the median OS was 176 months (95% CI: 136–240) in patients who did not receive radiation therapy and 158 months (95% CI: 120‐NA) in those who received radiation therapy. The 5‐ and 10‐year OS rates were 71.5% and 57.3% for patients who did not receive radiation therapy, and 78.1% and 59.0% for those who received radiation therapy, respectively. After PSM (1:1) matching, the median OS was 260 months (95% CI: 106 ~ NA) in the non‐radiation group and 163 months (95% CI: 123 ~ NA) in the radiation group. The 5‐year and 10‐year OS rates were 69.0% and 56.8% in the non‐radiation group and 78.9% and 61.5% in the radiation group, respectively.

### 3.3 Subgroup analysis

**FIGURE 3:**
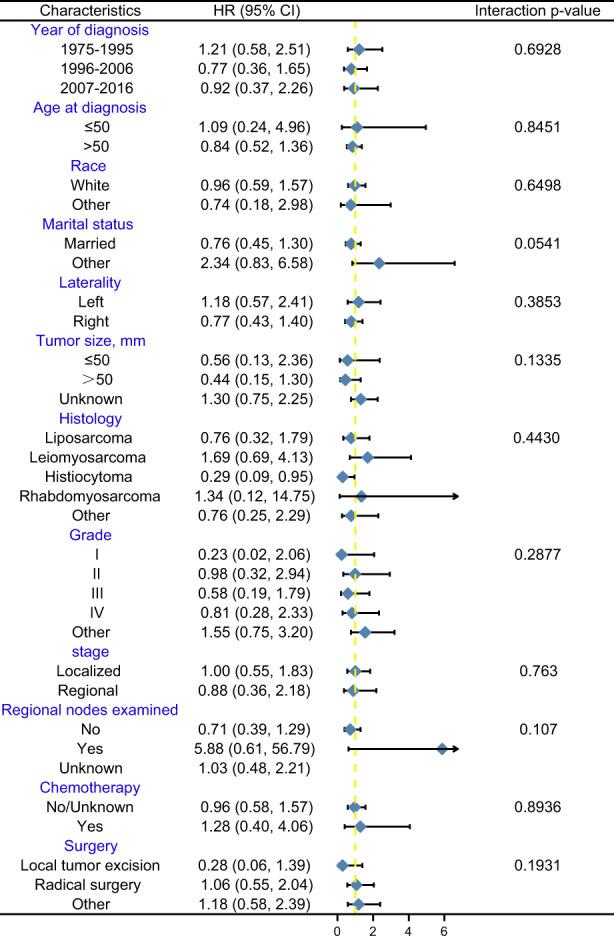
Forest plot showing subgroup analysis of spermatic cord tumor patients after PSM

We performed stratified analyses at different levels after PSM according to years of diagnosis, age, marital status, laterality, tumor size, tissue type, grade, stage, lymph node examination, chemotherapy, and surgery. The results are shown in Figure 3. The relationship between radiation therapy and OS remained stable across all subgroups, and the test for interaction was not statistically significant (all *p* > 0.05). The stratification analysis results before PSM were similar to those after PSM in Data S1.

### 3.4 Univariate and multivariate analysis

After PSM, univariate COX regression analysis showed that OS was significantly associated with years of diagnosis, age, laterality, histological type, grade, stage, lymph node examination, and surgical approach but not with radiotherapy. After including these meaningful variables and radiotherapy, multivariate COX analysis showed that age, histological type, grade, and stage were independent risk factors for OS in SCT patients, whereas radiotherapy was still not an independent risk factor for OS (HR 0.74, 95% CI 0.44–1.24, *p* = 0.25) (Table 2). The results of the univariate and multivariate COX regression analyses before PSM were consistent with those after PSM. Radiation therapy was also not an independent predictor of OS (Table S1).

**TABLE 2:** Univariate and multivariate analyses of OS in SCT patients after PSM

Characteristic	Univariable analysis		Multivariate analysis	
	HR (95% CI)	*p*‐value	HR (95% CI)	*p*‐value
Year of diagnosis				
1975–1995	Reference		Reference	
1996–2006	0.49 (0.28, 0.84)	**0.0094** *	2.75 (0.37, 20.41)	0.3233
2007–2016	0.52 (0.28, 0.98)	**0.0422** *	2.86 (0.36, 22.67)	0.3191
Age at diagnosis				
≤50	Reference		Reference	
>50	4.73 (2.16, 10.35)	**0.0001** *	5.15 (1.82, 14.57)	**0.002** *
Race				
White	Reference			
Other	0.75 (0.38, 1.46)	0.3986		
Marital status				
Married	Reference			
Other	0.70 (0.41, 1.18)	0.182		
Laterality				
Left	Reference		Reference	
Right	1.73 (1.08, 2.75)	**0.0213** *	1.54 (0.92, 2.57)	0.0987
Other	3.07 (0.41, 22.85)	0.2736	1.22 (0.11, 14.07)	0.8746
Tumor size, mm				
≤50	Reference			
>50	1.40 (0.59, 3.30)	0.4427		
Unknown	1.77 (0.83, 3.77)	0.1398		
Histology				
Liposarcoma	Reference		Reference	
Leiomyosarcoma	2.36 (1.29, 4.29)	**0.0051** *	1.63 (0.82, 3.21)	0.1611
Histiocytoma	3.81 (1.97, 7.38)	**<0.0001** *	2.30 (1.04, 5.09)	**0.0397** *
Rhabdomyosarcoma	0.84 (0.25, 2.82)	0.7765	2.47 (0.48, 12.68)	0.2802
Other	2.37 (1.20, 4.66)	**0.0125** *	1.61 (0.70, 3.72)	0.2627
Grade				
I	Reference		Reference	
II	5.04 (1.79, 14.14)	**0.0022** *	2.00 (0.61, 6.53)	0.25
III	3.65 (1.30, 10.24)	**0.0139** *	2.98 (0.98, 9.06)	**0.0542** *
IV	4.86 (1.75, 13.53)	**0.0024** *	3.25 (1.02, 10.40)	**0.0466** *
Other	5.07 (1.96, 13.11)	**0.0008** *	2.18 (0.70, 6.75)	0.1767
Stage				
Localized	Reference		Reference	
Regional	1.03 (0.61, 1.76)	0.9031	0.90 (0.51, 1.58)	0.7053
Distant	3.39 (1.34, 8.58)	**0.01** *	4.62 (1.51, 14.18)	**0.0074** *
Other	2.35 (0.99, 5.58)	0.0537	2.34 (0.91, 5.96)	0.076
Regional nodes examined				
No	Reference		Reference	
Yes	0.87 (0.31, 2.42)	0.7848	0.98 (0.30, 3.13)	0.9672
Unknown	2.63 (1.62, 4.26)	**<0.0001** *	1.63 (0.81, 3.31)	0.1736
Regional nodes positive				
No	Reference			
Yes	0.00 (0.00, Inf)	0.9948		
Unknown	0.97 (0.39, 2.43)	0.9554		
Radiation				
No	Reference		Reference	
Yes	0.949 (0.603, 1.492)	0.81944	0.74 (0.44, 1.24)	0.2487
Chemotherapy				
No/Unknown	Reference			
Yes	0.98 (0.53, 1.83)	0.9509		
Surgery				
Local tumor excision	Reference		Reference	
Radical surgery	1.24 (0.58, 2.67)	0.5837	0.79 (0.33, 1.85)	0.5813
Other	2.53 (1.15, 5.59)	**0.0214** *	2.61 (0.33, 20.62)	0.3621

Abbreviations: CI, Confidence intervals; HR, Hazard ratios; OS, Overall Survival; PSM, propensity score matching; SCT, Spermatic cord tumors.

* *p* < 0.05 was considered significant and marked in bold.

## 4 DISCUSSION

Although rare, SCT can seriously affect physical and mental health.^10^, ^11^, ^12^, ^13^ Urologists are not as familiar with the diagnosis and treatment of these types of tumors as they are of other primary testicular tumors; therefore, more research is needed.^2^ The clinical presentation of SCT is usually a groin or scrotal mass that grows slowly and painlessly.^1^, ^6^, ^14^, ^15^ For palpable masses, imaging evaluation using scrotal ultrasound,^16^ computed tomography,^17^ and magnetic resonance imaging^18^ are currently recommended. Currently, due to the scarcity of cases of malignant SCT, most treatment strategies refer to case reports, single‐institution series, literature reviews, and expert opinion.^5^ Radical resection remains the standard treatment option,^8^ while lymphatic dissection,^19^ radiation therapy,^20^ and chemotherapy^1^ remain controversial. Owing to the frequent recurrence of malignant SCT, long‐term follow‐up of patients is recommended.^9^, ^20^ Considering the resistance of SCT to chemotherapy, Rodríguez et al. do not recommend routine adjuvant chemotherapy, except for rhabdomyosarcoma.^5^ A study by Fagundes et al. showed that adjuvant radiation therapy reduced the risk of local recurrence after surgery.^10^ In this project, the largest study on the effect of radiotherapy on the overall survival rate of patients with malignant SCT was conducted based on SEER data.

From 1975 to 2016, 584 patients with SCT were included in the SEER database. For reliability of the results, a total of 389 patients with malignant SCT with a pathological diagnosis were included in this study after the exclusion of patients with multiple tumors and a survival time of less than 1 month (Figure 1). The mean age of these patients was 59.72 years, and the median survival time was 71 months (range, 32–150). Among the 389 patients, 324 (83.29%) were Caucasians. The numbers of cancer patients on the left 192 (49.36%) and the right 190 (48.84%) were similar. The most common pathological subtype was liposarcoma 203 (52.19%), followed by leiomyosarcoma 74 (19.02%). Among the grades, 124 (31.88%) were grade I (well differentiated). Grade II (moderately differentiated), grade III (poorly differentiated), and grade IV (undifferentiated; anaplastic) tumors accounted for approximately 12% of cases. Owing to the lack of a T (tumor) N(node) M (metastasis) staging system, we used the SEER historic stage A (1973–2015) system for description, with 252 (64.78%) localized, 90 (23.14%) regional, and 17 (4.37%) distant. These results are similar to those of the 2013 study of primary SCT by Rodríguez et al.^3^ via the SEER database (1973–2007). The trends found in this study remained unchanged over 10 years (Table 1).

The treatment method is the most important factor in malignant SCT and directly affects the patient's life expectancy and quality of life. The SEER database records patient surgery, chemotherapy, and radiotherapy information. Currently, the most poignant discussions have focused on the routine use of adjuvant radiotherapy for malignant SCT. Of the included cases, 285 (73.26%) did not receive radiotherapy and 104 (26.74%) received radiotherapy. Survival analysis showed no significant differences between the two groups (Figure 2A). The COX proportional hazards regression model showed that the radiotherapy strategy was not an independent risk factor for OS in patients with malignant spermatic tumor (Table S1). To exclude unbalanced features from affecting the results, we used PSM to balance the baseline features of the covariates (Table 1). After PSM, univariate and multivariate analyses showed that radiotherapy was not an independent factor affecting the overall survival rate of patients, but age, histological type, grade, and stage were. (Table 2). The Kaplan–Meier curve after PSM also showed no significant difference between the radiotherapy and non‐radiotherapy groups (Figure 2C).

Because most malignant SCT are sarcomas, the principles of surgical management of sarcomas, radical inguinal resection, and negative margins are followed.^5^ Currently, local recurrence after surgical resection is a major concern.^21^, ^22^, ^23^ Spermatic sarcoma spreads mainly through the inguinal canal to the groin into the peritoneal cavity; hematogenous and lymphatic invasion are less frequent.^14^ Regional lymphatic drainage of the spermatic cord includes ipsilateral pelvic, inguinal, common iliac, and paraaortic nodules.^24^, ^25^ Some investigators have proposed radiotherapy for these areas^26^; however, some investigators do not advocate radiotherapy for nodules because of the low propensity of nodules to spread.^9^, ^27^ Of the 389 patients included, only 35 (9.00%) underwent lymph node examination after surgical resection, and only 5 (1.29%) had positive lymph nodes (Table 1). Multivariate analysis after PSM also showed that lymph node status was not an independent risk factor in patients with malignant SCT (Table 2). As the SEER dataset does not have detailed records of lymph node dissection, our conclusions are limited. Hazariwala et al.^28^ reviewed the treatment results of 15 patients with spermatic sarcoma. Preoperative radiotherapy and complete resection are ideal treatment methods. Currently, there is no evidence of the efficacy of radiotherapy and chemotherapy before surgery for malignant tumors.^5^ Our cohort of patients who received preoperative radiotherapy was too small to determine whether it improved OS in malignant SCT. Some investigators recommend adjuvant radiation therapy for patients at a high risk of local recurrence.^29^ Ballo et al.^14^ reported durable local control observed in three patients who underwent surgery plus radiation. However, some investigators have reservations regarding adjuvant radiotherapy.^9^ Coleman et al.^6^ reported seven (50%) recurrences after adjuvant radiotherapy in 14 patients with malignant spermatic tumors, and no benefit of postoperative radiotherapy was observed. Similarly, survival outcomes were not significantly different between the 175 (68.09%) patients who underwent surgery and the 82 (31.90%) patients who underwent radiotherapy after surgery (Figure 2B). Our conclusions are limited due to the low incidence of malignant spermatic tumors and the limitations of the SEER database in providing the details of surgery and radiotherapy. We believe that the development of imaging and radiotherapy techniques and specific patient selection may aid better detection of the benefits of radiotherapy in patients with malignant SCT.

This study is the largest sample size used to analyze the effect of radiotherapy on the prognosis of malignant SCT. However, this study has some limitations.^1^ Preoperative radiotherapy and postoperative radiotherapy may have different outcomes, but the sample size was too small; therefore, we uniformly grouped them into the radiotherapy group.^2^ The SEER database lacks information on specific parameters affecting the effect of radiotherapy, such as the dose and exposure range.^3^ Owing to the large age span and involvement of multiple hospital cases, there are many different surgical methods.^4^ Information on chemotherapy in the SEER database was reported as “no/unknown” and “yes”. This also affects the analysis of the role of chemotherapy in patients with SCT.^5^ our study was a retrospective study based on the SEER database, and even with PSM, there is inevitably bias in patient selection, necessitating a prospective controlled clinical study to validate.

## 5 CONCLUSION

Based on the limited information in the SEER database, we concluded that radiotherapy had no significant advantage in improving the overall survival of patients with malignant spermatic tumors.

## AUTHOR CONTRIBUTIONS

**Yifu Liu:** Writing – original draft (equal). **zhicheng zhang:** Writing – original draft (equal). **Jinxiang wang:** Resources (equal); software (equal).

## FUNDING INFORMATION

No funding was received for this work.

## CONFLICT OF INTEREST

The authors declare that they have no competing interests.

## ETHICS APPROVAL AND CONSENT TO PARTICIPATE

This study used previously collected de‐identified data, and no additional authorization or ethical review was required.

## CONSENT FOR PUBLICATION

Not applicable.

## Supporting information


Data S1

Table S1

## Data Availability

The data are available on the Surveillance, Epidemiology, and End Results (SEER, http://seer.Cancer.gov) database.
