# Supplementary figures and images for: Effects of radiotherapy on the survival of patients with malignant spermatic cord tumors: A retrospective cohort study
Source: Cancer Med. 2022 Nov 10;12(5):5580–9. doi: 10.1002/cam4.5402 (PMC10028065; doi:10.1002/cam4.5402)

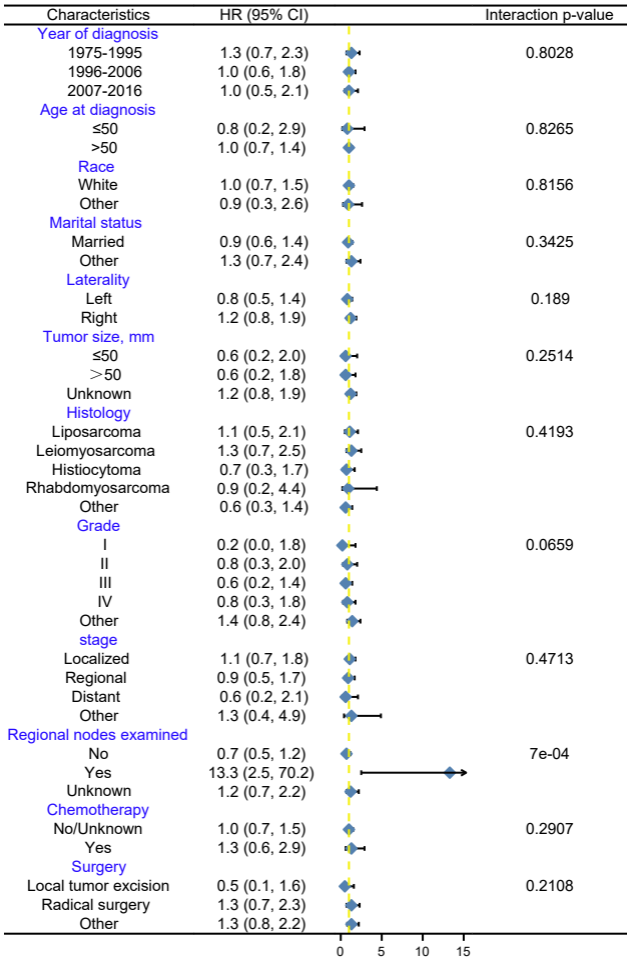

Supplement: Supplementary file 1 — Data S1 [file CAM4-12-5580-s001.pdf]
