# Supplementary material for: Effects of radiotherapy on the survival of patients with malignant spermatic cord tumors: A retrospective cohort study
Source: Cancer Med. 2022 Nov 10;12(5):5580–9. doi: 10.1002/cam4.5402 (PMC10028065; doi:10.1002/cam4.5402)
Supplement: Supplementary file 2 — Table S1 [file CAM4-12-5580-s002.docx]

**Table S1 |** Univariate and multivariate analyses of OS in SCT patients before PSM.

| **Characteristic** | **Univariable analysis** | | **Multivariate analysis** | |
| --- | --- | --- | --- | --- |
|  | **HR (95% CI)** | **P-value** | **HR (95% CI)** | **P-value** |
| **Year of diagnosis** |  |  |  |  |
| 1975-1995 | Reference |  | Reference |  |
| 1996-2006 | 0.6 (0.4, 0.9) | **0.009*** | 1.37 (0.72, 2.60) | 0.3422 |
| 2007-2016 | 0.6 (0.4, 0.9) | **0.028*** | 1.51 (0.69, 3.30) | 0.2992 |
| **Age at diagnosis** |  |  |  |  |
| ≤50 | Reference |  | Reference |  |
| >50 | 4.3 (2.6, 7.1) | **<0.001*** | 4.41 (2.32, 8.37) | **<0.0001*** |
| **Race** |  |  |  |  |
| White | Reference |  |  |  |
| Other | 0.8 (0.5, 1.2) | 0.299 |  |  |
| **Marital status** |  |  |  |  |
| Married | Reference |  |  |  |
| Other | 0.8 (0.6, 1.1) | 0.151 |  |  |
| **Laterality** |  |  |  |  |
| Left | Reference |  |  |  |
| Right | 1.1 (0.8, 1.5) | 0.398 |  |  |
| Other | 2.0 (0.9, 4.7) | 0.096 |  |  |
| **Tumor size, mm** |  |  |  |  |
| ≤50 | Reference |  |  |  |
| ＞50 | 0.9 (0.4, 1.7) | 0.654 |  |  |
| Unknown | 1.3 (0.8, 2.2) | 0.335 |  |  |
| **Histology** |  |  |  |  |
| Liposarcoma | Reference |  | Reference |  |
| Leiomyosarcoma | 2.7 (1.8, 4.0) | **<0.001*** | 2.30 (1.43, 3.72) | **0.0006*** |
| Histiocytoma | 3.6 (2.3, 5.7) | **<0.001*** | 2.28 (1.29, 4.01) | **0.0044*** |
| Rhabdomyosarcoma | 0.6 (0.3, 1.3) | 0.228 | 1.50 (0.58, 3.87) | 0.4015 |
| Other | 2.8 (1.8, 4.2) | **<0.001*** | 2.12 (1.24, 3.63) | **0.006*** |
| **Grade** |  |  |  |  |
| I | Reference |  | Reference |  |
| II | 3.1 (1.8, 5.2) | **<0.001*** | 1.58 (0.85, 2.94) | 0.1445 |
| III | 2.6 (1.5, 4.6) | **<0.001*** | 2.37 (1.25, 4.47) | **0.0078*** |
| IV | 3.0 (1.8, 5.2) | **<0.001*** | 2.22 (1.16, 4.26) | **0.0166*** |
| Other | 2.6 (1.6, 4.0) | **<0.001*** | 1.45 (0.82, 2.57) | 0.2021 |
| **stage** |  |  |  |  |
| Localized | Reference |  | Reference |  |
| Regional | 1.1 (0.8, 1.6) | 0.478 | 1.13 (0.77, 1.65) | 0.5422 |
| Distant | 4.8 (2.7, 8.5) | **<0.001*** | 7.49 (4.11, 13.64) | **<0.0001*** |
| Other | 3.1 (1.7, 5.4) | **<0.001*** | 2.51 (1.34, 4.71) | **0.0042*** |
| **Regional nodes examined** |  |  |  |  |
| No | Reference |  | Reference |  |
| Yes | 0.5 (0.2, 1.0) | 0.051 | 0.20 (0.02, 1.88) | 0.1591 |
| Unknown | 1.8 (1.3, 2.5) | **<0.001*** | 1.14 (0.75, 1.74) | 0.539 |
| **Regional nodes positive** |  |  |  |  |
| No | Reference |  |  |  |
| Yes | 0.8 (0.1, 6.8) | 0.861 | 0.45 (0.05, 4.00) | 0.4703 |
| Unknown | 2.3 (1.1, 4.9) | **0.031*** | 0.33 (0.04, 2.66) | 0.3003 |
| **Radiation** |  |  |  |  |
| No | Reference |  | Reference |  |
| Yes | 1.0 (0.7, 1.4) | 0.952 | 0.79 (0.54, 1.17) | 0.2466 |
| **Chemotherapy** |  |  |  |  |
| No/Unknown | Reference |  |  |  |
| Yes | 0.8 (0.5, 1.2) | 0.331 |  |  |
| **Surgery** |  |  |  |  |
| Local tumor excision | Reference |  | Reference |  |
| Radical surgery | 1.0 (0.6, 1.7) | 0.949 | 0.87 (0.50, 1.53) | 0.6284 |
| Other | 1.8 (1.1, 3.1) | **0.019*** | 1.90 (0.89, 4.06) | 0.0984 |

SCT, Spermatic cord tumors; OS, Overall Survival; HR, Hazard ratios; CI, Confidence intervals;

PSM, propensity score matching. *P < 0.05 was considered significant and marked in bold.

**Additional file 2 Table S1**
